# Cross-Species Identification of Urinary Small Extracellular Vesicle-Associated miR-223-3p as a Candidate Biomarker for Nephrolithiasis in a Discovery Cohort

**DOI:** 10.7150/ijms.136918

**Published:** 2026-08-24

**Authors:** Bo-Jung Chen, Jang Jeong Uk, Cheng-Huang Shen, Chiung-Yao Fang, Ya-Yan Lai, Yu-Wen Hong, Chi-Yang Eaw, Mei-Chiao Chuang, Jui-Chieh Chen

**Affiliations:** 1 Department of Urology, Ditmanson Medical Foundation Chiayi Christian Hospital, Chiayi 60002, Taiwan.; 2 Department of Medical Research, Ditmanson Medical Foundation Chiayi Christian Hospital, Chiayi 60002, Taiwan.; 3 Department of Biochemical Science and Technology, National Chiayi University, Chiayi 60004, Taiwan.

**Keywords:** Nephrolithiasis, Urinary small extracellular vesicle, microRNAs, biomarkers

## Abstract

**Background:**

Nephrolithiasis remains a major global health burden with limited non-invasive tools for early diagnosis and monitoring. Urinary small extracellular vesicle (sEV)-associated microRNAs (miRNAs) are stable molecular signatures that reflect renal pathophysiological shifts. This study aims to identify candidate urinary sEV-associated miRNAs for nephrolithiasis in a clinical discovery cohort and prioritize candidate biomarkers by integrating human urinary sEV miRNA profiles with publicly available murine nephrolithiasis datasets.

**Methods:**

Small RNA sequencing was performed on RNA isolated from urinary sEVs obtained from a clinical discovery cohort (25 patients with nephrolithiasis and 8 healthy controls) to identify candidate biomarkers associated with nephrolithiasis. Differentially expressed miRNAs (DEmiRNAs) were identified using a |log₂ fold change| > 1 and adjusted *P* < 0.05 threshold. Functional enrichment (GO/KEGG) was conducted on predicted target genes. To prioritize biologically relevant candidates, human DEmiRNAs were intersected with an available murine kidney miRNA dataset (GSE186793) and the ExoCarta database. The lead candidate, miR-223-3p, was further validated via RT-qPCR in human urinary sEV samples and urinary sEVs isolated from an independent CaOx-induced mouse model.

**Results:**

We identified 111 significantly dysregulated urinary sEV-associated miRNAs (105 upregulated, 6 downregulated) in patients with nephrolithiasis. Functional analysis linked these miRNAs to endocytosis, mitochondrial organization, and the MAPK/Wnt signaling pathways. Integrative analysis of the human urinary sEV, the murine kidney miRNA datasets (GSE186793), and the ExoCarta database prioritized a conserved five-miRNA signature (miR-223-5p, miR-223-3p, miR-342-3p, let-7i-5p, and miR-29c-3p). Among these candidates, miR-223-3p demonstrated the strongest translational potential and was therefore selected for further validation. RT-qPCR confirmed that miR-223-3p was significantly upregulated in both human urinary sEVs and urinary sEVs from the CaOx-induced mouse model.

**Conclusion:**

Our findings characterize a distinct urinary sEVs-associated miRNA landscape within this discovery cohort and identify miR-223-3p as a promising candidate biomarker for nephrolithiasis. Given the relatively small, clinically heterogeneous discovery cohort and the absence of an independent validation cohort, these findings should be considered hypothesis-generating and require validation in larger, independent, clinically stratified prospective studies before clinical application.

## Introduction

Nephrolithiasis, also commonly known as kidney stones, is a widespread and highly recurrent urological disorder characterized by the formation of solid deposits in the kidneys. According to current estimates, there is a variation in the prevalence of nephrolithiasis among different regions worldwide. The incidence of nephrolithiasis is approximately 5-10% in Europe, 4% in South America, and ranges from 1-19% in Asia 1, 2. The inter-country differences in nephrolithiasis prevalence can be attributed to diverse lithogenic factors, such as ethnicity, climate, diet, genetics, and metabolic disorders, etc. In Asia, the recurrence rate of urinary tract stones is around 21% to 53% after 3-5 years. The commonly formed stone components are calcium oxalate (CaOx) (75%-90%), followed by uric acid (5%-20%), calcium phosphate (6%-13%), ammonium magnesium phosphate (2%-15%), hydroxyapatite (1%), and cystine (0.5%-1%) 1. Despite advances in surgical management, the high recurrence rate and diverse etiologies of nephrolithiasis underscore the urgent need for improved mechanistic insights and effective preventive strategies 3.

In recent years, urinary small extracellular vesicles (sEVs) have increasingly been recognized as promising noninvasive biomarkers for nephrolithiasis. These nanosized extracellular vesicles encapsulate nucleic acids, proteins, and lipids, thereby reflecting the physiological and pathological status of renal cells. Their molecular cargoes remain stable in biofluids and exhibit disease-specific signatures, supporting their diagnostic potential in renal disorders 4-6.

Among urinary sEVs cargoes, microRNAs (miRNAs)—a class of small, non-coding RNA molecules typically 18-22 nucleotides in length—have emerged as pivotal post-transcriptional regulators of gene expression 7. Urinary sEV-associated miRNAs have been extensively investigated across a broad spectrum of renal diseases, underscoring their value as non-invasive biomarkers for diagnosis and mechanistic exploration. Previous studies have associated urinary sEV-associated miRNAs with idiopathic nephrotic syndrome, kidney injury, renal fibrosis, and nephrolithiasis, highlighting their potential clinical utility 8-13.

Although understanding of miRNAs derived from urinary sEVs in renal diseases has advanced, their specific regulatory roles in nephrolithiasis remain at an early stage. Notably, urinary sEV-associated miRNA profiles have not been characterized in Taiwanese populations, and the translational relevance of animal findings to human stone disease remains uncertain. To address these knowledge gaps, we conducted an exploratory discovery cohort study integrating urinary EV samples from Taiwanese patients with nephrolithiasis and a CaOx-induced murine model. Given the uncertainty regarding the interspecies consistency of urinary sEV-associated miRNAs, we first performed small RNA sequencing to identify differentially expressed miRNAs in the human cohort, followed by integrative bioinformatic analyses incorporating murine model data to prioritize translationally relevant candidates. This approach aims not only to develop population-specific biomarkers for Taiwanese patients but also to establish a cross-species molecular framework that supports translational validation, facilitating future evaluation of miRNA-targeted therapies and deeper elucidation of the molecular drivers of nephrolithiasis.

## Materials and methods

### Clinical sample collection and preprocessing

The study protocols were approved by the Institutional Review Board of Ditmanson Medical Foundation Chia-Yi Christian Hospital (IRB numbers: IRB2023077 and IRB098012). A total of 25 patients with different types of nephrolithiasis, including calcium oxalate, calcium phosphate, uric acid, struvite, and cystine stones, were enrolled as a discovery cohort, together with eight healthy individuals recruited as controls. Given the exploratory nature of this study, patients with heterogeneous clinical characteristics were included to facilitate initial biomarker discovery. Validation in larger, clinically stratified cohorts will be required in future studies. For each participant, 50 mL of midstream urine was collected in a sterile container. To minimize cellular contamination and preserve the integrity of urinary sEVs, urine samples were processed immediately using a stepwise differential centrifugation protocol. Samples were first centrifuged at 1,000 × g for 10 minutes at 4 °C to remove intact cells. The supernatant was then transferred and centrifuged at 3,200 × g for 10 minutes to eliminate cellular debris. The resulting cell-free urine supernatant was immediately stored at -80 °C until subsequent analysis.

### Isolation of urinary sEVs

Frozen urine samples were thawed and centrifuged at 3,200 × g for 15 minutes to remove residual debris. For all samples, a fixed input volume of 3 mL cell-free urine was used for urinary sEV isolation to ensure consistency across the study cohort. Three milliliters of the resulting supernatant were mixed thoroughly with an equal volume of Total Exosome Isolation Reagent (from urine) (Cat. No. 4484452; Thermo Fisher Scientific). The mixture was incubated overnight at 4 °C to allow precipitation of sEVs. Subsequently, samples were centrifuged at 21,100 × g for 1 hour at 4°C to pellet the sEV-enriched fraction. After complete removal of the supernatant, the sEV-enriched pellet was stored at -80 °C until further use.

### Total RNA extraction and quality assessment

Total RNA was extracted from isolated sEVs using TRIzol reagent (MRE-3200; EBL Biotechnology) and subsequently resuspended in 20 μL of DEPC-treated water. RNA purity was initially assessed using a NanoDrop spectrophotometer by measuring the absorbance ratios at 260/280 nm (A260/A280) and 260/230 nm (A260/A230) to ensure minimal protein and organic solvent contamination. The size distribution and integrity of small RNAs were further analyzed using the Fragment Analyzer 5200 system in conjunction with the Agilent DNF-471 RNA Kit (15 nt) (Agilent Technologies), enabling precise evaluation of small RNA enrichment to meet library preparation requirements. In addition, small RNA concentration was accurately quantified using Qubit microRNA Assay Kits (Thermo Fisher Scientific). The same quality control procedures were applied to all samples from both nephrolithiasis patients and healthy controls according to the manufacturer's recommendations prior to library preparation. Samples with sufficient small RNA quantity for library preparation, as determined by the Qubit microRNA Assay, were included for subsequent sequencing.

### Small RNA library construction and high-throughput sequencing

Five microliters of total RNA were used as the input material for small RNA library preparation using a commercially available small RNA library preparation kit using the QIAseq miRNA Library Kit (QIAGEN, Hilden, Germany). High-throughput sequencing was performed on the Illumina NovaSeq X Plus platform. Raw sequencing data were subjected to quality control processing, including removal of adapter sequences, low-quality reads, and reads with inappropriate length, to generate high-quality clean reads for subsequent analysis. All sequencing libraries were prepared and processed using the same experimental workflow to minimize potential batch effects.

### Bioinformatics and differential expression analysis

Raw miRNA read counts generated after alignment and feature counting were used as input for differential expression analysis using the DESeq2 package (version 1.44.0) in R (version 4.4.1). Differentially expressed miRNAs were identified based on an absolute log₂ fold change > 1 and an adjusted *p* value < 0.05. Multiple testing correction was performed using the Benjamini-Hochberg false discovery rate (FDR) procedure. For downstream visualization and classification analyses, including principal component analysis (PCA), hierarchical clustering heatmaps, and receiver operating characteristic (ROC) curve analysis, expression values were normalized as transcripts per million (TPM) to account for differences in library size. PCA was performed to evaluate intergroup separation and biological reproducibility among samples. The distribution of differentially expressed miRNAs was visualized using MA plots and volcano plots, while hierarchical clustering heatmaps were generated to illustrate the expression profiles of highly dysregulated miRNAs across individual samples.

### Functional enrichment and pathway analysis

To predict the potential biological functions of differentially expressed miRNAs, target genes were identified using the multiMiR framework, which integrates multiple databases. Predicted target genes of significantly upregulated and downregulated miRNAs were analyzed separately. Gene Ontology (GO) enrichment analysis was performed to classify enriched terms into biological process, molecular function, and cellular component categories. In addition, pathway enrichment analysis was conducted based on the Kyoto Encyclopedia of Genes and Genomes (KEGG) database to elucidate the key signaling pathways potentially regulated by the identified miRNAs.

### Diagnostic performance evaluation (ROC curve analysis)

To assess the potential of urinary sEV-associated miRNAs as noninvasive diagnostic biomarkers for nephrolithiasis, receiver operating characteristic (ROC) curves were constructed for 15 significantly upregulated candidate miRNAs based on their TPM expression levels. The area under the ROC curve (AUC) was calculated to evaluate the sensitivity and specificity of each miRNA in discriminating patients with nephrolithiasis from healthy controls.

### Cross-species conservation analysis

To enhance the translational relevance of our findings, differentially expressed miRNAs identified from human urinary sEVs were subjected to a cross-species comparative analysis with the following datasets: (1) the miRNA expression profile derived from renal tissues of a CaOx-induced mouse nephrocalcinosis model (GSE186793, which represents the only publicly available murine miRNA dataset related to nephrolithiasis at the time of this study); and (2) the list of murine (*Mus musculus*) sEV-associated miRNAs curated in the ExoCarta database. Because no publicly available urinary sEV miRNA sequencing dataset from murine nephrolithiasis models was identified after a comprehensive search of public repositories, GSE186793 was utilized solely for candidate prioritization during the initial bioinformatic screening rather than to infer direct biological equivalence between kidney tissue and urinary sEVs. Venn diagram analysis was performed to identify candidate miRNAs with concordant differential expression trends across the human and murine datasets, thereby providing a rational basis for subsequent experimental validation using urinary sEVs from both human patients and the CaOx-induced mouse model.

### Mouse model of nephrolithiasis

Ten 8-week-old male BALB/c mice were used to establish the CaOx nephrolithiasis model and were randomly assigned to a control group or a CaOx group (n = 5 per group). The CaOx group received drinking water containing 0.75% (v/v) ethylene glycol and 0.5% (w/v) ammonium chloride for 30 days to induce calcium oxalate nephrolithiasis, whereas the control group received normal drinking water. Harvested kidneys were fixed in 4% paraformaldehyde (PFA), dehydrated through a 10-30% sucrose gradient, embedded in optimal cutting temperature (OCT) compound, and sectioned into 5 μm frozen sections. Hematoxylin and eosin (H&E) staining was performed to evaluate morphological alterations, including tubular dilation. Calcium salt deposition was assessed by Von Kossa staining with ultraviolet light exposure for 4 hours. In addition, mouse urine samples were collected, and total RNA was isolated from urinary sEVs using the same reagents and TRIzol-based extraction protocol as applied to human samples to ensure cross-species comparability for miRNA validation analyses.

### Reference miRNA stability analysis

Candidate endogenous reference miRNAs were initially identified from the small RNA sequencing data based on their minimal differential expression between nephrolithiasis patients and healthy controls (log₂FC ≈ 0). To further determine whether these candidate miRNAs exhibited sufficient expression stability for RT-qPCR normalization, their stability was subsequently evaluated using three complementary algorithms: geNorm, NormFinder, and BestKeeper. In addition, the distribution of raw Ct values was examined to further assess expression consistency across all human urinary sEV samples.

### RT-qPCR validation

To validate the sequencing results, total RNA isolated from urinary sEVs was reverse-transcribed with the Mir-X miRNA First-Strand Synthesis Kit (Takara Bio, USA) following the manufacturer's instructions. Quantitative PCR was performed on the MyGo PCR Detection System using the Mir-X miRNA qRT-PCR SYBR kit (Takara Bio). Reactions were performed with miRNA-specific forward primers and the kit-provided mRQ 3′ universal reverse primer. miR-320a-3p was selected as the endogenous reference miRNA for RT-qPCR normalization based on the reference miRNA stability analysis. The specific forward primers for the target and internal control miRNAs were: 5'-TGTCAGTTTGTCAAATACCCCA-3' (miR-223-3p), 5'-TGTAGTGTTTCCTACTTTATGGA-3' (miR-142-3p), and 5'-AAAAGCTGGGTTGAGAGGGCGA-3' (miR-320a-3p). Relative expression levels were calculated using the 2^-ΔΔCt^ method.

### Association of miR-223-3p expression with clinical characteristics

To evaluate potential confounding effects of patient background characteristics on urinary sEV-derived miR-223-3p expression, correlation analyses were performed between miR-223-3p expression and age, body mass index (BMI), and urinary pH. In addition, urinary sEV-derived miR-223-3p expression was compared between urine culture-positive and urine culture-negative patients to assess the potential influence of concomitant urinary tract infection.

### Statistical analysis

All raw sequencing read counts were normalized to transcripts per million (TPM). Clinical characteristics were summarized as mean ± standard deviation (SD) or median with interquartile range (IQR), as appropriate. Comparisons of categorical variables between groups (e.g., sex) were performed using the chi-square test. Differential expression analysis was performed using the DESeq2 package in R based on raw miRNA read counts. Statistical significance was determined using the Benjamini-Hochberg false discovery rate (FDR) correction, with an adjusted *p* value < 0.05 and |log₂ fold change| > 1 considered significant. Spearman's rank correlation analysis was performed to evaluate the associations between urinary sEV-derived miR-223-3p expression and clinical variables. Differences in urinary sEV-derived miR-223-3p expression between urine culture-positive and urine culture-negative patients were analyzed using the Mann-Whitney U test. The diagnostic performance of candidate biomarkers was evaluated using receiver operating characteristic (ROC) curve analysis, and the area under the curve (AUC) was calculated to assess sensitivity and specificity. ROC curve analysis was performed using TPM-normalized expression values. Because this study was designed as an exploratory discovery cohort with a relatively small sample size, no resampling or cross-validation was performed. All bioinformatic analyses and data visualizations, including PCA, volcano plots, and heatmaps, were performed using the R environment using TPM-normalized expression values for visualization purposes. Functional enrichment analysis was conducted using the MultiMiR framework in conjunction with the Gene Ontology and KEGG databases, with *p* < 0.05 considered indicative of statistically significant enrichment.

## Results

### Distinctive expression profiles of urinary sEV-associated miRNAs in nephrolithiasis patients

A total of 25 patients with nephrolithiasis and 8 healthy controls were enrolled in this clinical discovery cohort. The clinical characteristics of the study participants are summarized in Table 1. The mean age of patients in the nephrolithiasis group was 58.2 years, with 68% male (n = 17) and 32% female (n = 8) participants. There was no significant difference in sex distribution between the patient and control groups (*p* = 0.70). The mean body mass index (BMI) of patients was 27.8 kg/m², and the mean urinary pH was 6.34. Regarding stone composition, calcium oxalate stones were the most prevalent (52%), followed by struvite stones (28%) and calcium carbonate stones (12%). Stone locations varied, with renal calculi accounting for 52%, while upper ureteral stones (20%) and staghorn calculi (20%) were also observed. Additionally, 36% of patients demonstrated positive urine cultures, with the predominant pathogens being *Escherichia coli* and *Proteus mirabilis*. Because this study was designed as a discovery cohort, the heterogeneous clinical characteristics—including different stone compositions and the presence of positive urine cultures in a subset of patients—were retained to capture the diversity of patients encountered in clinical practice rather than to perform subtype-specific analyses. Before small RNA sequencing, urinary sEV-enriched fractions were characterized by Western blotting. Representative EV markers (CD9, TSG101, and HSP70) were readily detected, whereas the endoplasmic reticulum marker calnexin was absent. Comparable protein loading was confirmed by Ponceau S staining, indicating successful enrichment of urinary sEVs suitable for downstream analyses (Supplementary Figure S1). To investigate the global expression landscape of urinary sEV-associated miRNAs in nephrolithiasis, small RNA sequencing was performed on urinary sEVs isolated from 25 nephrolithiasis patients and 8 healthy controls. Principal component analysis (PCA) based on transcripts per million (TPM) values revealed a clear separation between the two groups, with PC1 and PC2 accounting for 36% and 17% of the total variance, respectively (Figure 1A). Differential expression analysis identified 111 significantly dysregulated miRNAs, defined by |log₂ fold change| > 1 and adjusted *p*-value < 0.05, including 105 upregulated and 6 downregulated miRNAs in patients (Figure 1B). The MA and volcano plots illustrated the magnitude and statistical significance of these expression changes (Figures 1B and 1C), with red dots denoting miRNAs that met both biological and statistical thresholds. Notably, several candidate miRNAs exhibited both high abundance and a marked increase in fold change. This initial screening highlights the potential of these highly dysregulated sEV-associated miRNAs as promising biomarkers and mechanistic targets for further validation in nephrolithiasis.

### Functional enrichment analysis of differentially expressed urinary sEV-associated miRNAs

To elucidate the potential biological roles of the dysregulated urinary sEV-associated miRNAs in nephrolithiasis, we performed separate functional enrichment analyses on the predicted target genes of significantly upregulated and downregulated miRNAs using the MultiMiR framework. For the 27 miRNAs that were significantly upregulated (log₂ fold change > 4), Gene Ontology (GO) enrichment analysis revealed strong enrichment in biological processes such as “regulation of protein catabolic process,” “endocytosis,” and “mitochondrial membrane organization,” as well as molecular functions like “GTPase regulator activity” and “DNA-binding transcription factor binding.” Cellular component terms included “extracellular exosome” and “nucleoplasm” (Figure 2A). KEGG pathway analysis of the same miRNAs indicated significant enrichment in signaling cascades such as “Proteoglycans in cancer,” “Hippo signaling pathway,” “Wnt signaling pathway,” and “MAPK signaling pathway,” all of which are involved in cellular proliferation, apoptosis, and tissue remodeling (Figure 2B). Conversely, analysis of the six significantly downregulated miRNAs (log₂ fold change < -1) revealed target gene enrichment in GO terms such as “ubiquitin-dependent protein catabolic process,” “autophagy,” “RNA localization,” and “histone deacetylase binding,” suggesting involvement in intracellular transport and epigenetic regulation (Figure 2C). KEGG pathway analysis further indicated that these downregulated miRNAs are associated with key pathways including “Autophagy - animal,” “Axon guidance,” “Focal adhesion,” and “Colorectal cancer,” which may reflect a loss of protective cellular stress responses in nephrolithiasis pathology (Figure 2D). Collectively, these functional enrichment results highlight the distinct biological processes and signaling networks regulated by the upregulated and downregulated sEV-associated miRNAs in nephrolithiasis patients, offering mechanistic insights and potential therapeutic targets.

### Highly upregulated miRNAs identified as potential diagnostic biomarkers in nephrolithiasis

To identify miRNAs that are significantly upregulated in nephrolithiasis, we performed differential expression analysis between patients and healthy controls. A total of 15 miRNAs with a log₂ fold change (log₂FC) greater than 5 and an adjusted p-value < 0.05 were selected for visualization. As shown in Figure 3, the heatmap illustrates the expression profiles of these miRNAs across individual samples. Although hsa-miR-16-5p, hsa-miR-143-3p, and hsa-miR-93-5p exhibited the highest absolute expression levels (TPM) among the upregulated miRNAs, their fold changes were relatively modest (log₂FC = 5.57, 6.07, and 5.28, respectively) compared to other candidates in the panel. The intensity of red coloration in the heatmap reflects the expression level measured in TPM, with deeper shades indicating higher abundance. Notably, hsa-miR-142-3p demonstrated the most substantial upregulation (log₂FC = 12.08), followed by hsa-miR-92a-3p, hsa-miR-142-5p, and hsa-miR-200b-3p, suggesting that these miRNAs may play critical roles in the pathogenesis of nephrolithiasis. To further evaluate their diagnostic potential, receiver operating characteristic (ROC) curve analysis was performed using TPM values derived from small RNA sequencing data. As shown in Figure 4, all 15 selected miRNAs exhibited strong discriminatory power in differentiating nephrolithiasis patients from healthy controls. Among them, hsa-miR-142-3p achieved the highest diagnostic performance with an area under the curve (AUC) of 0.9800, followed by hsa-miR-143-3p and hsa-miR-93-5p (AUC = 0.9600 for both). Several other miRNAs, including hsa-miR-223-3p, hsa-miR-15b-5p, hsa-miR-28-5p, and hsa-miR-142-5p, also achieved AUC values greater than 0.90, indicating excellent sensitivity and specificity. Collectively, these findings identify several urinary sEV-associated miRNAs as candidate diagnostic biomarkers that warrant further validation in independent cohorts.

### Cross-species identification of conserved urinary sEV-associated miRNAs associated with nephrolithiasis in humans and mice

Murine models of nephrolithiasis remain widely used tools for investigating the pathogenesis of nephrolithiasis and evaluating potential biomarkers and therapeutic strategies. However, the translational applicability of findings derived from animal models to human disease remains a persistent challenge. To address this, we intersected differentially expressed miRNAs identified by small RNA sequencing of urinary sEVs from nephrolithiasis patients with two additional sources: (1) kidney tissue miRNA expression profiles from a CaOx-induced nephrocalcinosis mouse model (GSE186793), and (2) sEV-associated miRNAs cataloged for *Mus musculus* in the ExoCarta database. Applying a threshold of |log₂ fold change| > 1 and adjusted *p*-value < 0.05, we identified five miRNAs—miR-223-5p, miR-223-3p, miR-342-3p, let-7i-5p, and miR-29c-3p—that overlapped across all three datasets (Figure 5). Notably, four of these miRNAs exhibited consistent upregulation in both human and murine datasets, while miR-29c-3p was upregulated in human samples but downregulated in the mouse model. These findings highlight the existence of shared sEV-associated miRNA signatures that may contribute to the pathophysiology of nephrolithiasis, and support the broader translational utility of murine models in elucidating disease mechanisms, identifying candidate biomarkers, and evaluating therapeutic strategies.

### Evaluation of Endogenous Reference miRNAs for RT-qPCR Normalization

Candidate endogenous reference miRNAs were initially selected from the small RNA sequencing dataset based on their minimal differential expression between nephrolithiasis patients and healthy controls. Among these candidates, miR-320a-3p exhibited virtually no change in expression (log₂FC = -7.02 × 10⁻⁶) and was therefore selected for subsequent stability evaluation. To rigorously evaluate the stability of the selected candidate reference miRNAs, geNorm, NormFinder, and BestKeeper analyses were performed. geNorm analysis ranked miR-320a-3p among the most stable candidates based on its low M value (Figure 6A). Similarly, NormFinder identified miR-320a-3p as a highly stable candidate with a low stability value (Figure 6B). BestKeeper analysis further demonstrated that miR-320a-3p exhibited favorable expression stability across the study cohort (Figure 6C). Although slight differences in the exact rankings were observed among the three algorithms, miR-320a-3p consistently demonstrated favorable stability across all three analyses. Furthermore, analysis of raw Ct values showed minimal variation in miR-320a-3p expression across all human urinary sEV samples, providing additional support for its suitability as the endogenous reference miRNA for subsequent RT-qPCR normalization (Figure 6D).

### *In Vivo* validation of candidate miRNAs using an CaOx-induced mouse model of nephrolithiasis

To evaluate the clinical relevance of the candidate biomarkers, miR-223-3p was selected for further validation. RT-qPCR analysis was performed using total RNA isolated from urinary sEVs in the clinical cohort. The results demonstrated that urinary sEV-derived miR-223-3p levels were significantly elevated in patients with nephrolithiasis compared with healthy controls after normalization to miR-320a-3p, which was identified as the most stable endogenous reference miRNA through geNorm, NormFinder, and BestKeeper analyses (Figure 7A). To evaluate whether urinary sEV-derived miR-223-3p expression was influenced by patient background characteristics, Spearman's rank correlation analyses were performed between miR-223-3p expression and age, BMI, and urinary pH (Table 2). No significant correlations were observed with age (ρ = -0.033, *p* = 0.877), BMI (ρ = -0.184, *p* = 0.379), or urinary pH (ρ = 0.146, *p* = 0.487), indicating that miR-223-3p expression was not significantly associated with these clinical variables in the present cohort. Because a subset of patients exhibited positive urine cultures, we further compared urinary sEV-derived miR-223-3p expression between urine culture-positive and urine culture-negative patients to assess the potential confounding effect of concomitant urinary tract infection. As shown in Table 3, no significant difference in miR-223-3p expression was observed between the two groups (median [IQR]: 1337.67 [487.61-21105.86] vs. 1136.54 [505.94-5843.51], respectively; Mann-Whitney U test, *p* = 0.978). These findings suggest that the elevated urinary sEV-derived miR-223-3p expression observed in nephrolithiasis patients is unlikely to be primarily attributable to differences in age, BMI, urinary pH, or urine culture status within this discovery cohort. Subsequently, to confirm these findings in an* in vivo* setting, we established a CaOx-induced mouse model of nephrolithiasis. Histopathological analysis (Figure 7B) confirmed successful stone formation, with H&E staining showing tubular dilation and Von Kossa's staining revealing significant calcium deposits. Consistent with our clinical observations, miR-223-3p expression was also significantly upregulated in the urinary sEVs of the nephrolithiasis mice compared to the control group (Figure 7C). Together with the clinical correlation analyses, these findings support that increased urinary sEV-derived miR-223-3p expression is associated with nephrolithiasis rather than with the examined patient background characteristics or urine culture status. These findings support miR-223-3p as a candidate non-invasive biomarker, based on its strong concordance between human patients and the murine model.

## Discussion

Nephrolithiasis arises from complex processes involving biomineralization and cellular stress responses. Recent studies have demonstrated that EVs are dynamic participants in renal physiological and pathological regulation. The molecular cargo of EVs is determined by the microenvironment of their donor cells and can mediate intercellular communication that regulates water and solute transport, organ development, and renal hemodynamics. Accumulating evidence suggests that EVs play dual roles in nephrolithiasis, as urinary EVs contain stone-inhibitory factors that suppress CaOx crystal growth, aggregation, and adhesion to renal epithelial cells under physiological conditions but also contribute to disease progression under pathological conditions 14-16. However, under pathological conditions, EV-mediated signaling promotes inflammatory responses and tissue remodeling, thereby contributing to the progression of nephrolithiasis 17, 18.

During the early stages of stone formation, miRNAs within extracellular vesicles play a critical role in regulating mineral homeostasis. Previous studies have shown that miR-148b-5p contained in urinary extracellular vesicles can suppress the expression of the calcitonin receptor (Calcr) through the circRNA-83536/miR-24-3p regulatory axis, thereby contributing to the development of hypercalciuria and calcium stone formation 11. In addition, specific tRNA-derived small RNAs (tsRNAs), such as tRF-Lys-TTT-5005c, are significantly elevated in patients with CaOx stones. These molecules promote osteogenic-like transformation through activation of the BMP signaling pathway, thereby accelerating the formation of Randall's plaques 19. Collectively, these molecular alterations illustrate how extracellular vesicles can precondition the microenvironment at the genetic regulatory level, creating conditions favorable for crystal nucleation and deposition.

As crystal formation progresses, injured renal tubular epithelial cells (RTECs) and immune cells establish an inflammatory propagation network via EVs. miR-93-3p has been shown to induce macrophage polarization toward the pro-inflammatory M1 phenotype and promote the formation of macrophage extracellular traps (METs), thereby exacerbating interstitial injury 19. Concurrently, RTECs damaged by oxalate secrete EVs containing Ambra1 protein, which trigger ferroptosis and autophagy in neighboring healthy cells, establishing a deleterious feed-forward loop 20. This process is also accompanied by endoplasmic reticulum stress and lysosomal dysfunction in macrophages, prompting the release of EVs enriched with GRP94 that exhibit high binding affinity. These EVs further activate inflammatory responses and neutrophil chemotaxis, ultimately accelerating renal tissue injury and promoting the pathological progression of kidney stone formation 17, 21.

Given the critical role of EVs in the pathological process, modulation of EV-mediated signaling has emerged as a promising therapeutic strategy. A previous report has demonstrated that EVs derived from adipose-derived stem cells carrying miR-20b-3p can effectively alleviate CaOx-induced renal injury and suppress stone formation by regulating ATG7-mediated autophagy and the TLR4-dependent inflammatory pathway 22. In the field of natural therapeutics, lemon-derived exosome-like vesicle nanoparticles (LEVNs) have exhibited remarkable renal-targeting capability and can effectively halt the progression of stone pathology by antagonizing endoplasmic reticulum stress 23. Collectively, these findings suggest that both exogenous miRNA-based EV engineering and plant-derived biomimetic nanoparticles provide a novel dimension for precision strategies aimed at preventing kidney stone recurrence.

miR-223-3p has recently emerged as a critical molecular switch in the progression of renal pathology, exhibiting significant protective and regulatory functions across multiple kidney disease models. Notably, miR-223-3p plays multifaceted roles in attenuating renal inflammation and oxidative stress. Accumulating evidence has identified the NLRP3 inflammasome as a central downstream target of miR-223-3p. In models of renal ischemia-reperfusion injury (RIRI) and sepsis-associated acute kidney injury (SAKI), upregulation of miR-223-3p suppresses NLRP3 expression, thereby inhibiting endothelial pyroptosis and reducing the release of pro-inflammatory cytokines 24, 25. Furthermore, the transcription factor KLF6 has been shown to repress miR-223-3p promoter activity, leading to activation of the NLRP3/Caspase-1 signaling pathway, which indirectly corroborates the protective role of miR-223-3p against sepsis-induced renal injury 26.

Beyond direct modulation of the inflammasome, miR-223-3p contributes to the maintenance of renal homeostasis through diverse signaling pathways. In diabetic kidney disease (DKD), miR-223-3p targets IL6ST and suppresses STAT3 signaling, thereby mitigating endothelial dysfunction 27. In acute kidney injury (AKI), bone marrow mesenchymal stem cell (BMSC)-derived exosomes enriched in miR-223-3p have been reported to target HDAC2 and promote SNRK expression, exerting anti-inflammatory and anti-pyroptotic effects 28. Importantly, loss of miR-223-3p function or its sequestration by competing endogenous RNAs, such as circVmn2r1, accelerates tubular epithelial cell senescence and promotes renal aging 29. Collectively, these findings underscore the essential role of miR-223-3p in preserving renal structural integrity and functional homeostasis.

In nephrolithiasis, A previous report has shown that suppression of lncRNA XIST attenuates CaOx-induced inflammation and oxidative stress by enhancing the miR-223/NLRP3 axis 30. Consistent with previous reports, our clinical data demonstrated that miR-223-3p was markedly upregulated in patients with nephrolithiasis. Together, these observations suggest that miR-223-3p may participate in inflammatory responses during renal injury, although its functional role in nephrolithiasis remains to be experimentally established. Importantly, because miR-223-3p is known to be associated with innate immune responses and myeloid cell activation, we further evaluated potential clinical confounding factors. No significant associations were observed between urinary sEV-derived miR-223-3p expression and age, BMI, or urinary pH. Furthermore, miR-223-3p expression did not differ significantly between urine culture-positive and urine culture-negative patients. Although the limited sample size precludes definitive exclusion of infection-related effects, these findings suggest that the elevated urinary sEV-derived miR-223-3p observed in this cohort is unlikely to be primarily explained by patient background characteristics or concomitant urinary tract infection. The precise functional role of miR-223-3p in nephrolithiasis remains to be experimentally validated. One possible explanation is that the increased urinary sEV-derived miR-223-3p expression represents an endogenous response to crystal-induced tubular epithelial injury and the accompanying inflammatory processes. Nevertheless, this hypothesis requires direct experimental validation.

Therefore, our findings support the potential of urinary sEV-derived miR-223-3p as a non-invasive biomarker for nephrolithiasis. Whether miR-223-3p directly regulates NLRP3 signaling or other downstream pathways in nephrolithiasis requires further functional investigation. Another key contribution of this study is the systematic evaluation of miR-320a-3p as a stable endogenous reference miRNA for RT-qPCR normalization of urinary sEV-associated miRNAs in this human discovery cohort. Small RNA sequencing revealed that both exhibited minimal log2FC variation and abundant expression in human nephrolithiasis samples. Normalization using either reference consistently demonstrated a significant upregulation of miR-223-3p in both human and murine models. Compared with conventional controls such as U6, which may vary under pathological conditions, this data-driven, cross-species-validated approach minimizes technical variability and improves quantification accuracy. Collectively, these findings not only highlight the role of miR-223-3p in nephrolithiasis but also establish a robust framework for urinary sEV-associated miRNA analysis, supporting its development as a non-invasive clinical biomarker.

This study has several limitations. First, this investigation represents a discovery-stage study with a relatively small sample size, a limited number of healthy controls, and no independent validation cohort. Therefore, the findings should be regarded as hypothesis-generating. Second, the heterogeneous study population and limited sample size precluded robust subgroup analyses according to stone subtype or infection status, although no significant association was observed between urinary sEV-derived miR-223-3p expression and urine culture status. Third, the chemically induced CaOx mouse model may not fully recapitulate the chronic progression of human nephrolithiasis, and the proposed biological mechanisms of miR-223-3p were not directly validated. Finally, urinary miRNA expression was normalized using miR-320a-3p and a fixed urine input volume rather than urinary creatinine concentration or EV particle number. Future multicenter clinical cohorts and mechanistic studies are needed to validate the diagnostic and biological significance of urinary sEV-derived miR-223-3p.

Overall, our findings characterize the urinary sEV-associated miRNA landscape in nephrolithiasis and identify miR-223-3p as a promising candidate biomarker. The absence of significant associations with the evaluated clinical variables in this discovery cohort further supports its potential as a urinary sEV-based biomarker. Future studies using larger, independent, and clinically stratified cohorts will be essential to validate its diagnostic performance and clinical applicability.

## Supplementary Material

Supplementary figure and methods.

## Figures and Tables

**Figure 1:**
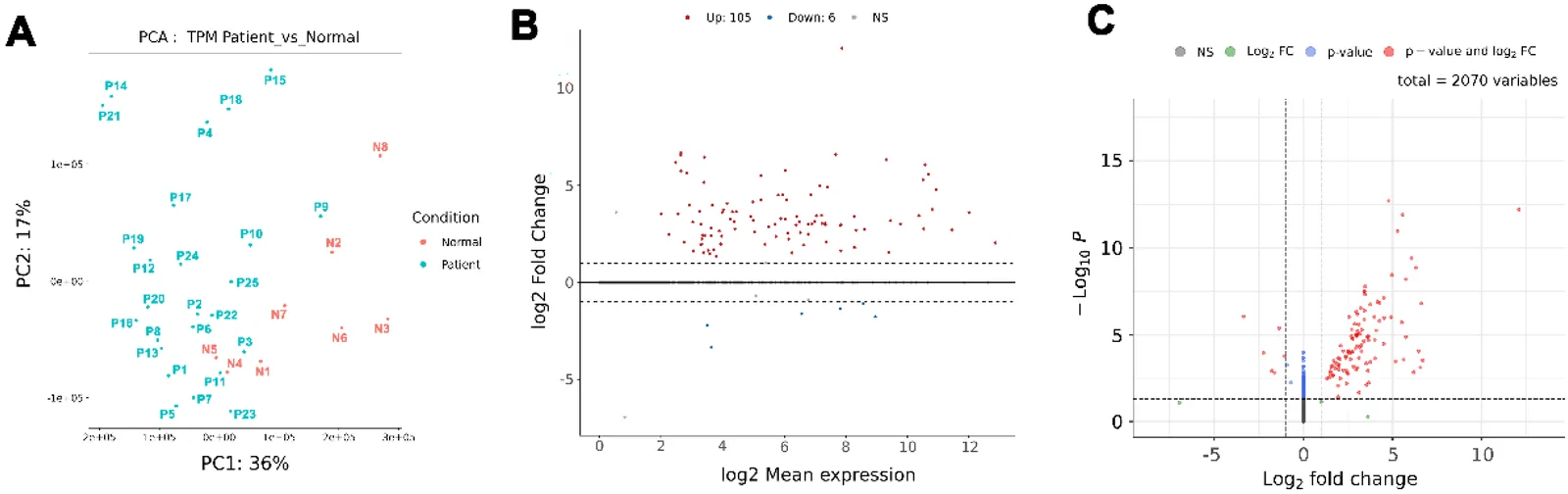
** Principal component analysis and differential expression analysis of urinary sEV-associated miRNAs from nephrolithiasis patients and healthy controls.** (A) PCA based on TPM values of sEV-associated miRNAs illustrates the separation between nephrolithiasis patients (blue, n = 25) and healthy controls (red, n = 8). (B) MA plot showing log₂ fold change (y-axis) versus log₂ mean expression (x-axis), with red and blue dots indicating significantly upregulated and downregulated miRNAs, respectively, in patients. (C) Volcano plot displaying differential expression of all detected miRNAs (n = 2,070). The x-axis represents the log₂ fold change between nephrolithiasis patients and healthy controls, and the y-axis represents the -log₁₀ *p*-value. Dashed lines indicate thresholds for statistical significance (-log₁₀ *p* > 1.3, equivalent to *p* < 0.05) and biological relevance (|log₂ fold change| > 1). Red dots indicate miRNAs that meet both criteria.

**Figure 2:**
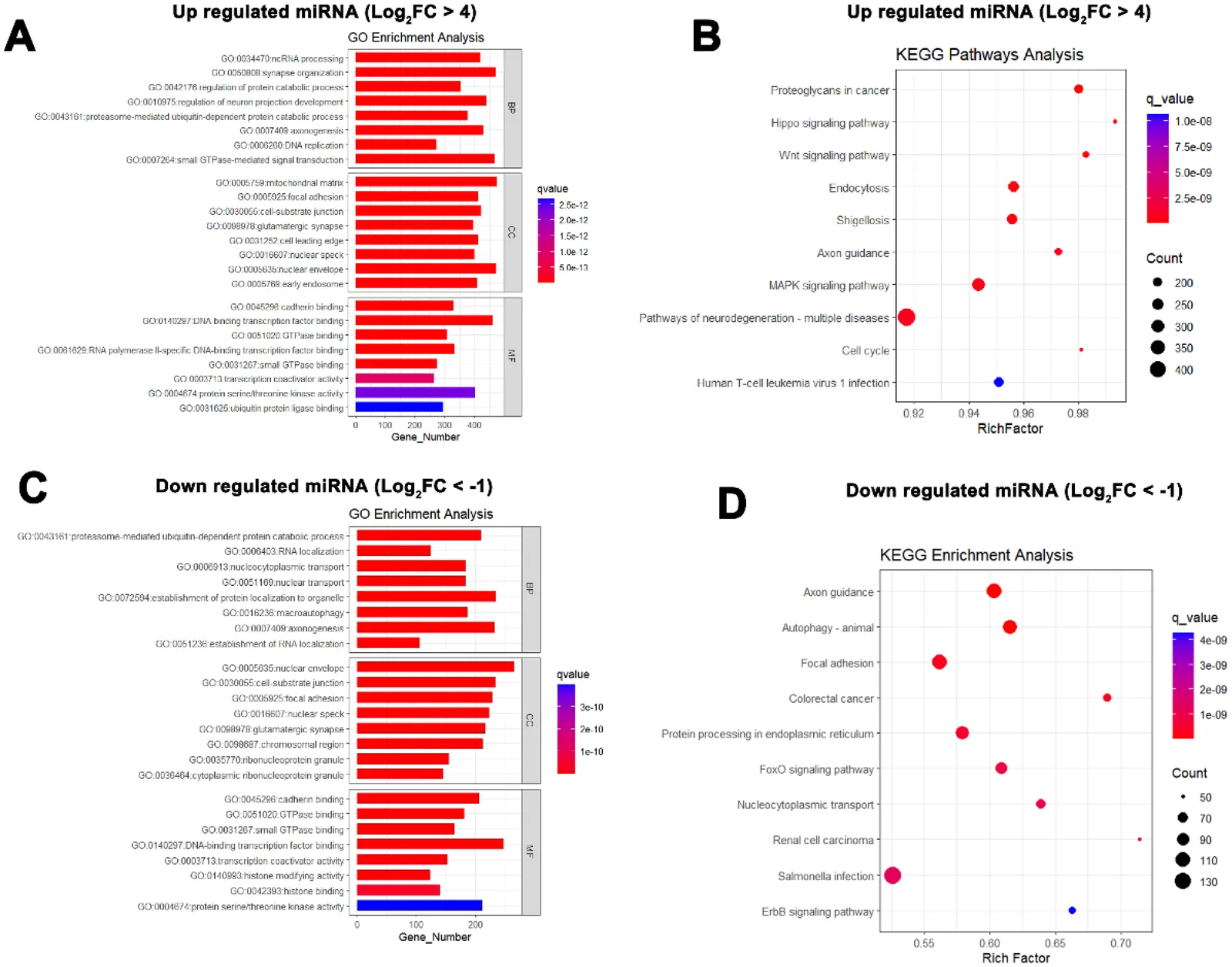
** Functional enrichment analysis of predicted target genes of differentially expressed miRNAs in nephrolithiasis patients.** (A) GO enrichment analysis of upregulated miRNAs. The bar plot displays the top enriched GO terms—comprising biological processes, cellular components, and molecular functions—based on the predicted target genes of 27 significantly upregulated miRNAs (log₂ fold change > 4) in nephrolithiasis patients. The x-axis represents the number of genes associated with each GO term, and the color gradient indicates the statistical significance (q-value). (B) KEGG pathway enrichment analysis of upregulated miRNAs. The bubble plot shows significantly enriched KEGG pathways targeted by the same 27 upregulated miRNAs. The x-axis represents the enrichment score (Rich Factor), bubble size reflects the number of genes involved, and the color scale corresponds to the q-value. (C) GO enrichment analysis of downregulated miRNAs. The bar plot presents the top enriched GO terms based on the predicted target genes of 6 significantly downregulated miRNAs (log₂ fold change < -1) in nephrolithiasis patients. The x-axis indicates the number of associated genes, and bar color represents the q-value. (D) KEGG pathway enrichment analysis of downregulated miRNAs. The bar plot illustrates the significantly enriched KEGG pathways based on the predicted target genes of the 6 downregulated miRNAs. The x-axis represents the number of genes involved, and the color gradient indicates statistical significance (q-value).

**Figure 3:**
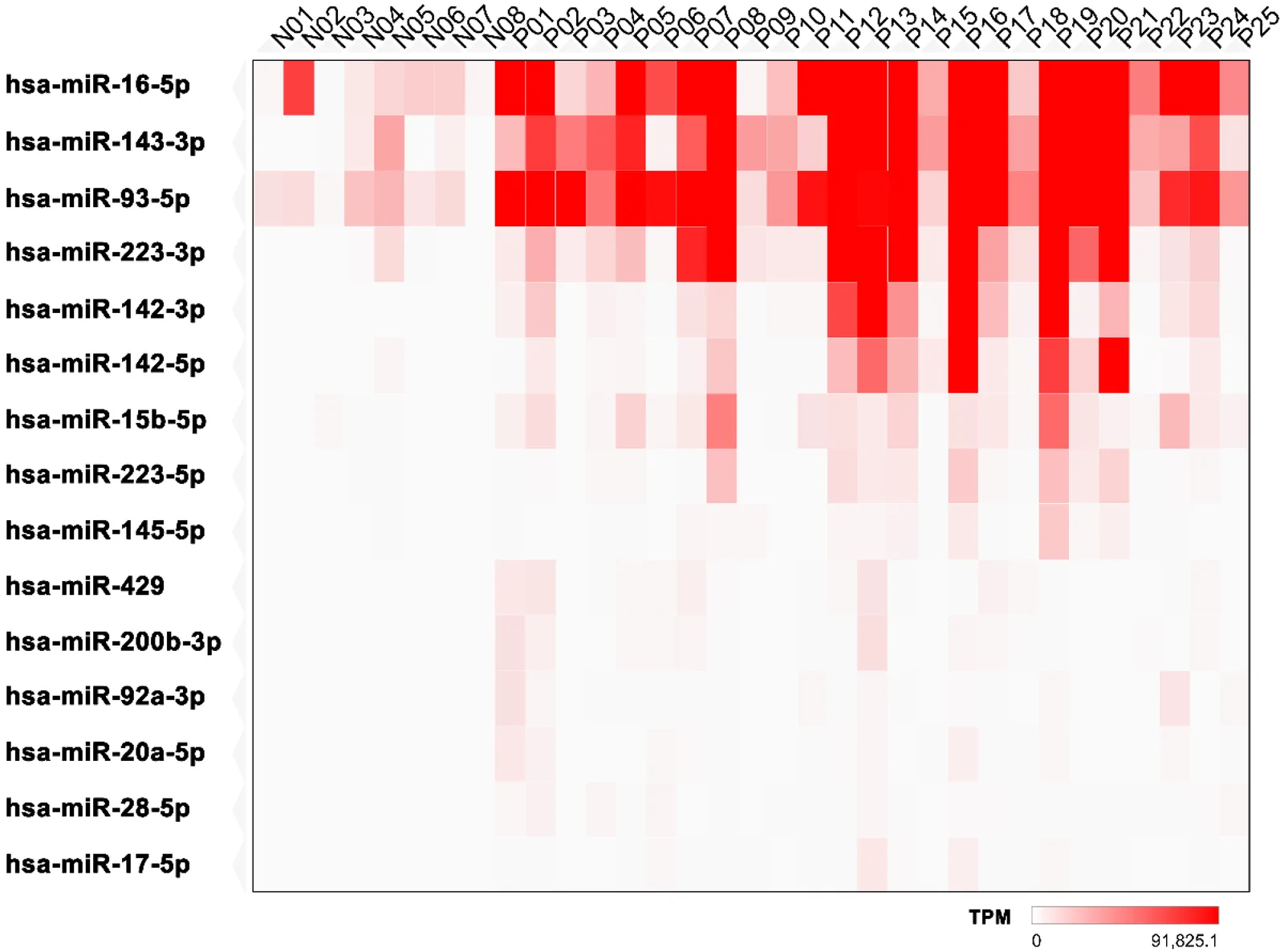
** Heatmap of significantly upregulated miRNAs in nephrolithiasis patients.** The heatmap illustrates the expression profiles of significantly upregulated miRNAs in nephrolithiasis patients compared to healthy controls. Only miRNAs with a log₂ fold change > 5 and *p*adj < 0.05 were included. Color intensity represents expression levels measured in TPM, with deeper red indicating higher expression.

**Figure 4:**
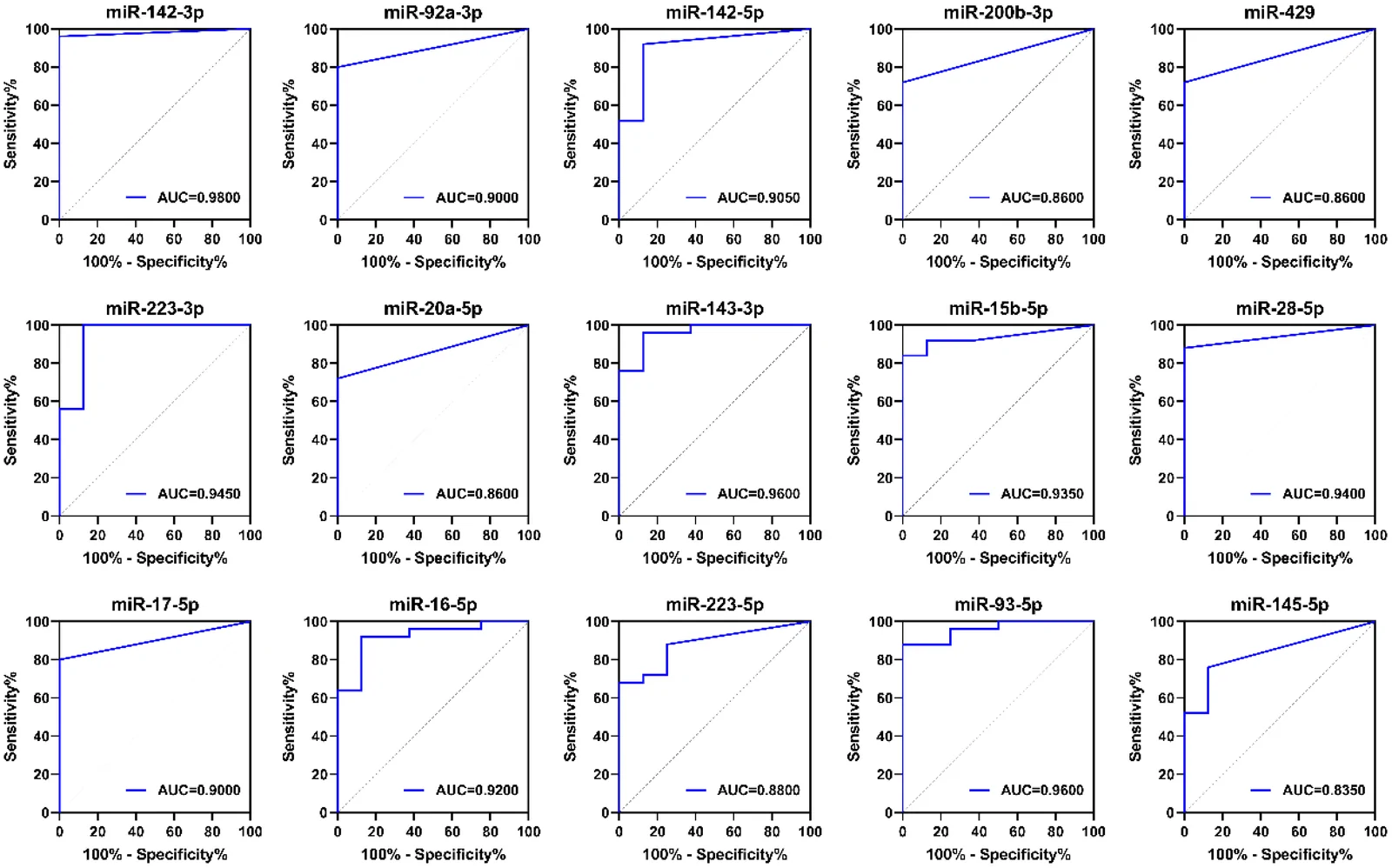
** ROC curve analysis of selected upregulated urinary sEV-associated miRNAs in nephrolithiasis patients.** ROC curve analysis was conducted to evaluate the diagnostic performance of 15 miRNAs that were significantly upregulated in urinary sEVs from patients with nephrolithiasis compared with healthy controls. miRNAs were selected based on small RNA sequencing results with log₂ fold change > 5 and *p*adj < 0.05. The expression data used for ROC analysis were derived from TPM values obtained by small RNA sequencing. Each panel shows the ROC curve of an individual miRNA, with its corresponding AUC value shown. Higher AUC values indicate stronger discriminatory power in distinguishing patients from controls.

**Figure 5:**
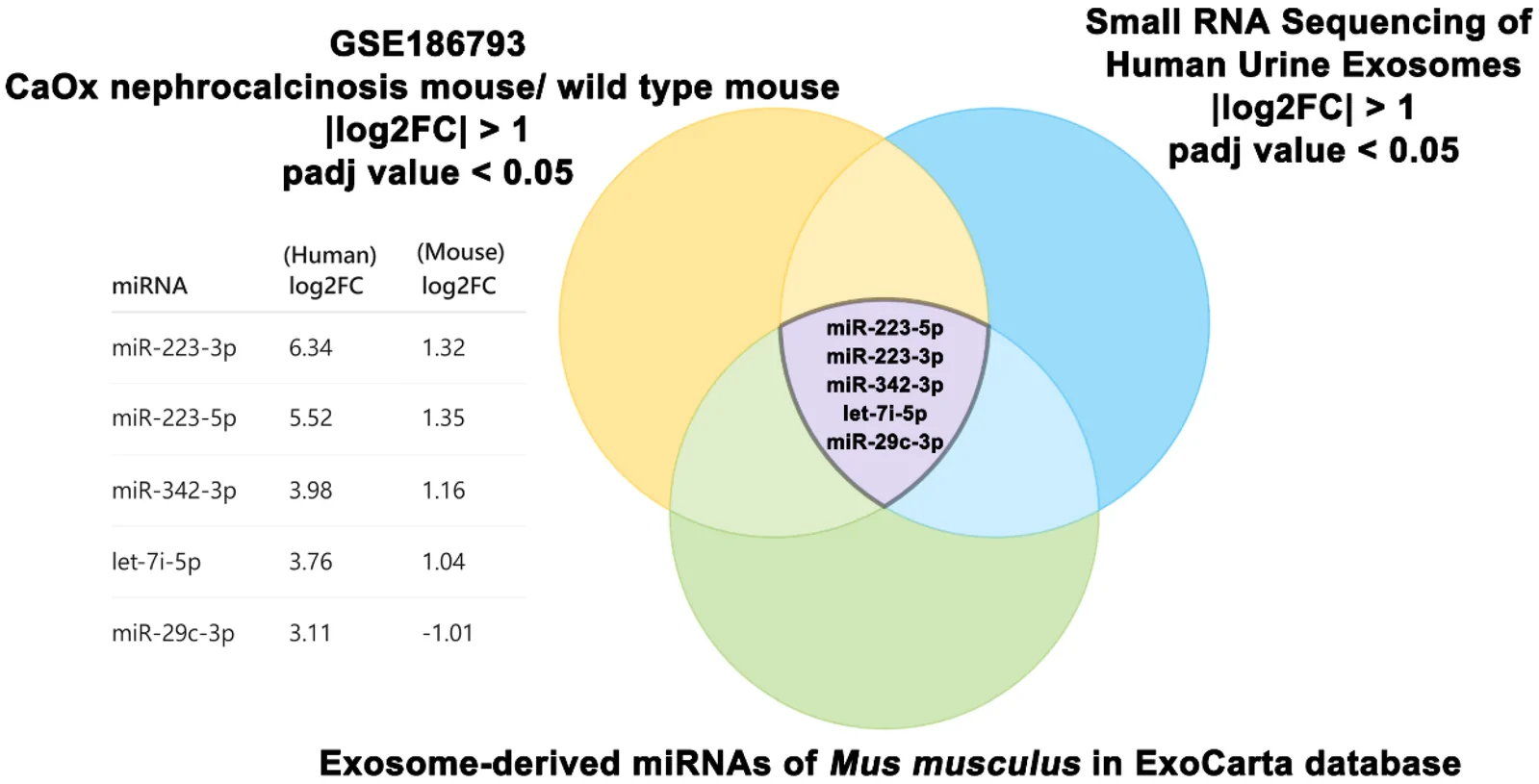
** Overlapping differentially expressed sEV-associated miRNAs in human and mouse nephrolithiasis models.** Venn diagram illustrating the intersection of differentially expressed miRNAs (|log₂ fold change| > 1, padj < 0.05) from three sources: (1) small RNA sequencing of urinary sEVs from patients with nephrolithiasis compared with healthy controls, (2) a mouse model of CaOx-induced nephrocalcinosis (GSE186793; diseased vs. wild-type mice), and (3) sEV-associated miRNAs of *Mus musculus* reported in the ExoCarta database. Five miRNAs—miR-223-5p, miR-223-3p, miR-342-3p, let-7i-5p, and miR-29c-3p—were commonly identified across all datasets. The table lists their log₂ fold changes in human and mouse datasets, demonstrating consistent differential expression across the human discovery cohort and the murine dataset.

**Figure 6:**
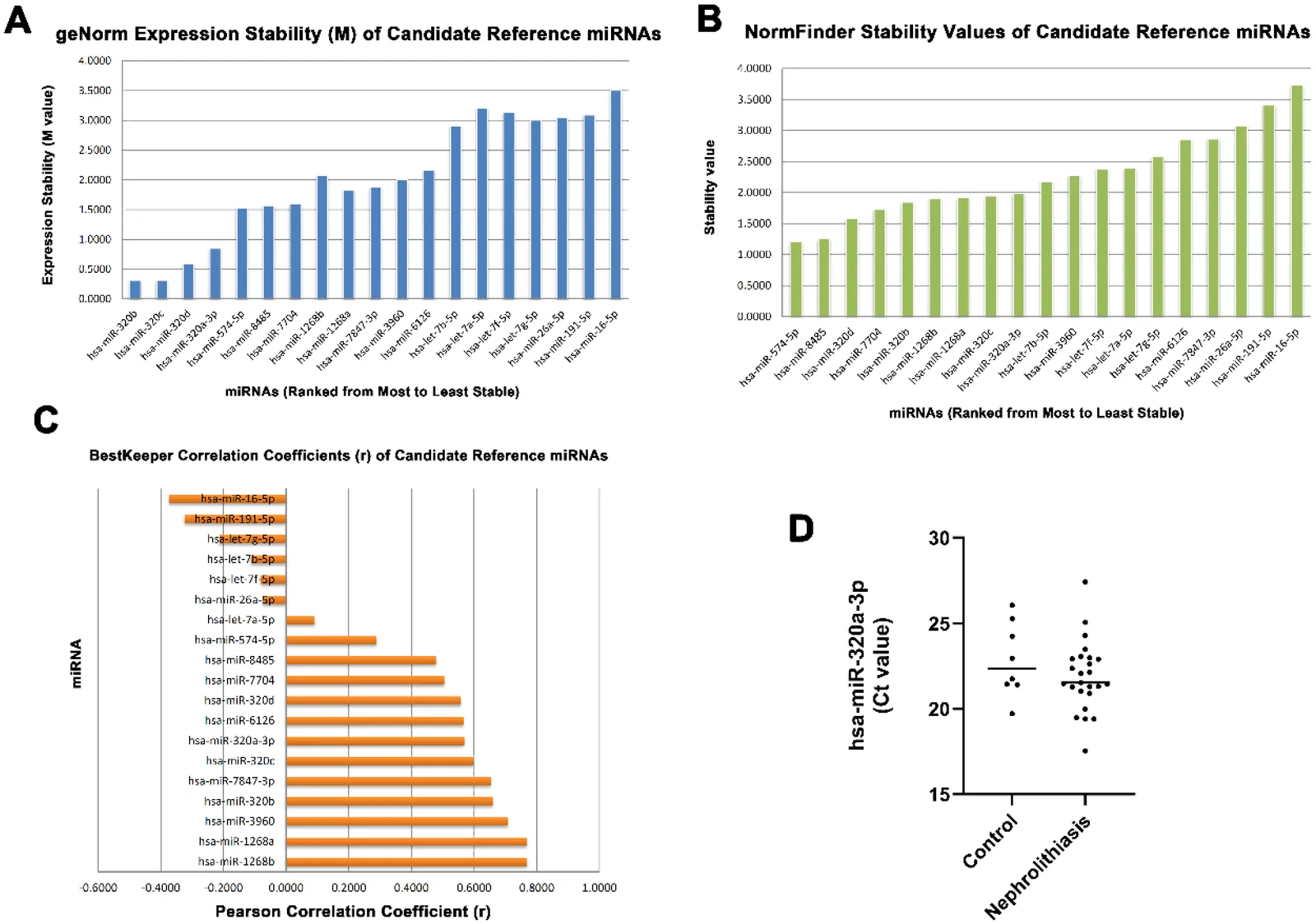
** Systematic selection and stability evaluation of candidate endogenous reference miRNAs.** Candidate endogenous reference miRNAs identified from the human urinary sEV small RNA sequencing dataset were systematically evaluated using the geNorm, NormFinder, and BestKeeper algorithms and RT-qPCR. (A) geNorm expression stability (M) values, where lower M values indicate greater expression stability. (B) NormFinder stability values, where lower values indicate greater expression stability. (C) BestKeeper Pearson correlation coefficients (r), where higher correlation coefficients indicate greater expression stability. (D) Ct value distribution of hsa-miR-320a-3p in healthy controls (n = 8) and nephrolithiasis patients (n = 25), showing no significant difference between groups (Mann-Whitney U test, *p* > 0.05).

**Figure 7:**
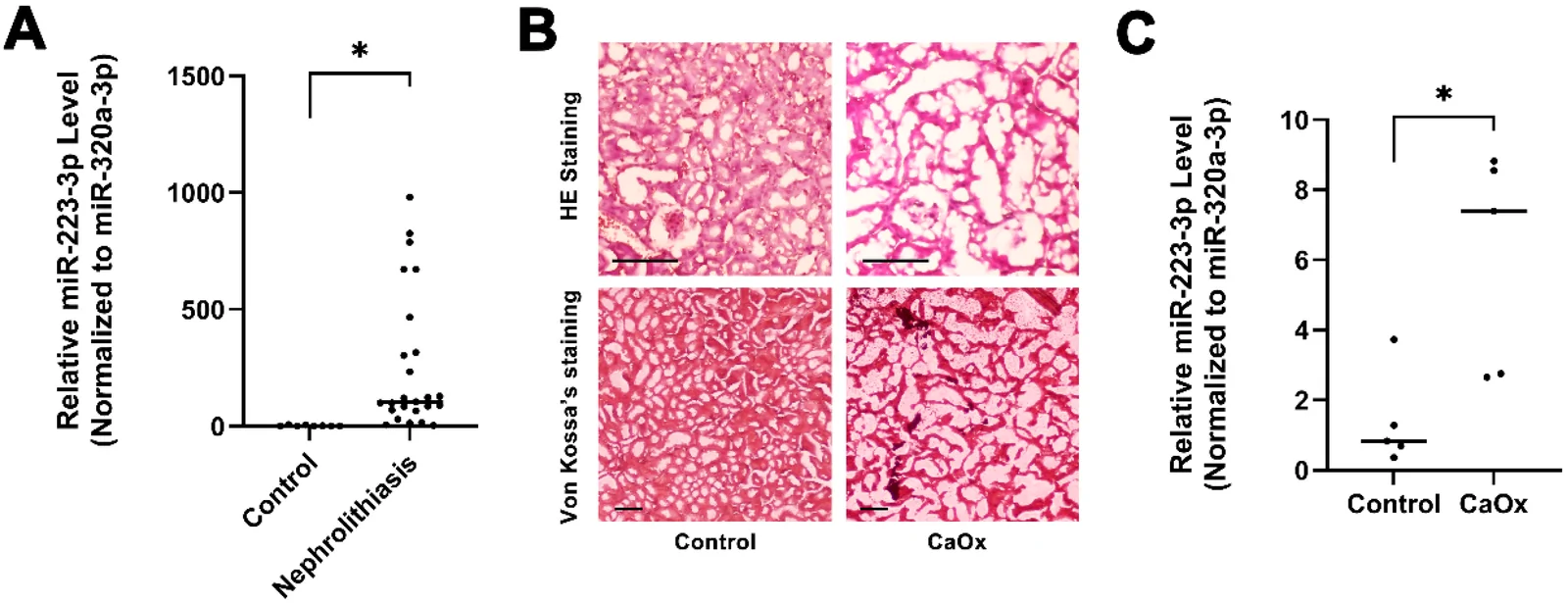
** Validation of miR-223-3p as a potential biomarker in clinical nephrolithiasis and a CaOx-induced murine model.** (A) Expression of miR-223-3p in human urinary sEVs. Relative levels of miR-223-3p were determined by qPCR in urinary sEVs from healthy controls and patients with nephrolithiasis. Data were normalized to miR-320a-3p to ensure robustness. Each data point represents an individual subject; horizontal bars indicate the median. (B) Histopathological assessment of the CaOx-induced mouse model. Representative micrographs of kidney sections from control mice and mice challenged with CaOx. Upper panels: Hematoxylin and Eosin (H&E) staining showing tubular dilation and structural alterations in the CaOx group. Lower panels: Von Kossa's staining highlighting significant black calcium crystal deposits in the renal parenchyma of CaOx-induced mice. Scale bars = 10 µm. (C) Upregulation of miR-223-3p in the murine nephrolithiasis model. qPCR analysis confirmed the significant elevation of miR-223-3p in the urinary sEVs of CaOx-induced mice compared to the control group, consistent with clinical findings. Results are presented using normalization standards (miR-320a-3p).

**Table 1:** Demographic and clinical characteristics of patients with kidney stones and healthy controls

Variable	Patients (n = 25)	Controls (n = 8)	*p*-value
Age (years)			
Mean±SD	58.2 ± 8.9	49.6 ± 7.1	—
Median (IQR)	59(52-65)	51.5 (48.8-53.3)	—
Range	41-75	36-58	—
Sex (Male/Female)	n (%)		
	Male 17 (68%)Female 8 (32%)	Male 4 (50%) Female 4 (50%)	0.70 (Chi-square test)
BMI (kg/m²)			
Mean±SD	27.8 ±4.3	23.3 ± 2.6	—
Median (IQR)	26.9(24.6-30.1)	23.5 (22.8-24.3)	—
Range	21.4-35.8	18-27	—
Urine pH			
Mean±SD	6.34 ±0.77	—	—
Median (IQR)	6.5 (5.5-7.0)	—	—
Range	5.0 - 8.0	—	—
Stone type	n (%)		
Calcium oxalate	13(52%)	—	—
Struvite (Ammonium magnesium phosphate)	7(28%)	—	—
Calcium carbonate	3(12%)	—	—
Uric acid	1(4%)	—	—
Mixed / Other	1(4%)	—	—
Cystine	0(0%)	—	—
Calcium phosphate	0(0%)	—	—
Total	25(100%)	—	—
Stone Location	n (%)		
Renal stone	13 (52%)	—	—
Upper ureteral stone	5 (20%)	—	—
Staghorn stone	5 (20%)	—	—
Renal pelvic stone	1 (4%)	—	—
Bilateral stones	1 (4%)	—	—
Urine Culture	n (%)		
Positivity	9 (36%)	—	—
Negative	16 (64%)	—	—
Top 3 bacteria	1. Escherichia coli2. Proteus mirabilis3. Klebsiella pneumoniae	—	—
Other isolates	Streptococcus mitis, Candida albicans, Morganella morganii	—	—

**Table 2:** Correlation between urinary sEV-derived miR-223-3p expression and clinical characteristics of patients with nephrolithiasis

Variable	Spearman's ρ	P value
Age (years)	-0.033	0.877
Body Mass Index (BMI)	-0.184	0.379
Urinary pH	0.146	0.487

Note. Correlations were assessed using Spearman's rank correlation analysis (n = 25). Statistical significance was defined as P < 0.05.

**Table 3:** Comparison of urinary sEV-derived miR-223-3p expression according to urine culture status in patients with nephrolithiasis

Urine culture status	n	miR-223-3p expression, Median (IQR)	P value
Negative	16	1136.54 (505.94-5843.51)	0.978
Positive	9	1337.67 (487.61-21105.86)	

Note: Data are presented as median (interquartile range, IQR) (n = 25). Differences between groups were analyzed using the Mann-Whitney U test Statistical significance was defined as *p* < 0.05.
