# Supplementary material for: Cross-Species Identification of Urinary Small Extracellular Vesicle-Associated miR-223-3p as a Candidate Biomarker for Nephrolithiasis in a Discovery Cohort
Source: Int J Med Sci. 2026 Aug 24;23(10):3169–82. doi: 10.7150/ijms.136918 (PMC13617494; doi:10.7150/ijms.136918)
Supplement: Supplementary file 1 [file ijmsv23p3169s1.pdf]

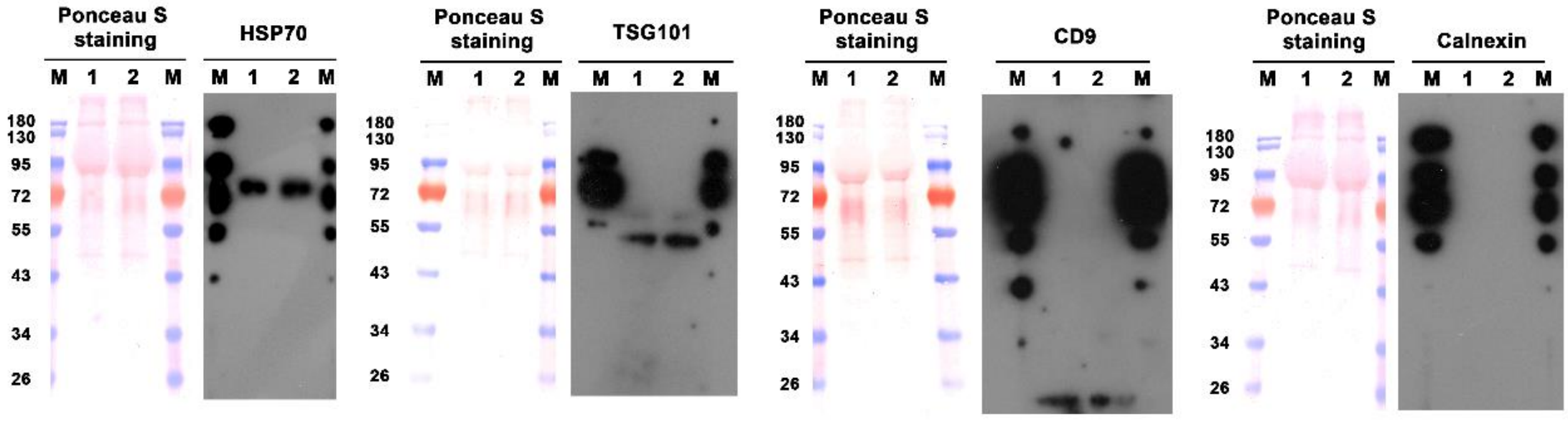

Supplementary Figure S1. Western blot characterization of urinary sEV-enriched fractions isolated from two independent healthy donors. Representative EV-associated proteins (CD9, TSG101, and HSP70) were detected, whereas the endoplasmic reticulum marker calnexin was not detected. Ponceau S staining was used to verify comparable protein loading and transfer efficiency.

Supplementary Materials and Methods

Western blot characterization of urinary sEV-enriched fractions

Urinary small extracellular vesicle (sEV)-enriched fractions were isolated from urine samples collected from two independent healthy donors using the same isolation procedure described in the main Materials and Methods. Total protein was extracted from the isolated urinary sEV-enriched fractions, and 30 μg of protein per sample was separated on 12% SDS–polyacrylamide gels (SDS–PAGE) and subsequently transferred onto polyvinylidene fluoride (PVDF) membranes. Membranes were stained with Ponceau S to verify comparable protein loading and transfer efficiency prior to immunoblotting.

The membranes were blocked with 5% non-fat milk in Tris-buffered saline containing 0.1% Tween-20 (TBST) for 1 h at room temperature and incubated overnight at 4°C with the following primary antibodies: anti-CD9 (Abcam, ab263019), anti-TSG101 (Abcam, ab125011), anti-HSP70 (Abcam, ab181606), and anti-calnexin (Abcam, ab133615). After washing with TBST, membranes were incubated with the appropriate horseradish peroxidase (HRP)-conjugated secondary antibodies for 1 h at room temperature. Protein bands were visualized using enhanced chemiluminescence (ECL) reagents and captured with a chemiluminescence imaging system.

CD9, TSG101, and HSP70 were used as positive markers for urinary sEVs, whereas calnexin served as a negative marker for intracellular contamination. The presence of EV-associated proteins together with the absence of calnexin confirmed successful enrichment of urinary sEVs with minimal cellular contamination.
